# Patient and public involvement and engagement in the ASCEND PLUS trial: reflections from the design of a streamlined and decentralised clinical trial

**DOI:** 10.1186/s13063-024-08393-2

**Published:** 2024-08-22

**Authors:** Muram El-Nayir, Rohan Wijesurendra, David Preiss, Marion Mafham, Leandros Tsiotos, Sadman Islam, Anne Whitehouse, Sophia Wilkinson, Hannah Freeman, Ryonfa Lee, Wojciech Brudlo, Genna Bobby, Bryony Jenkins, Robert Humphrey, Amy Mallorie, Andrew Toal, Elnora C. Barker, Dianna Moylan, Graeme Thomson, Firoza Davies, Hameed Khan, Ian Allotey, Susan Dickie, John Roberts

**Affiliations:** 1 Clinical Trial Service and Epidemiological Studies Unit, Nuffield Department of Population Health, University of Oxford, Oxford, OX3 7LF UK; 2 Public Advisory Group; ASCEND PLUS Clinical Trial, Nuffield Department of Population Health, University of Oxford, Oxford, OX3 7LF UK

**Keywords:** PPIE, Patient and public involvement and engagement, Diabetes, Clinical trial

## Abstract

**Introduction:**

ASCEND PLUS is a randomised controlled trial assessing the effects of oral semaglutide on the primary prevention of cardiovascular events in around 20,000 individuals with type 2 diabetes in the UK. The trial’s innovative design includes a decentralised direct-to-participant invitation, recruitment, and follow-up model, relying on self-completion of online forms or telephone or video calls with research nurses, with no physical sites. Extensive patient and public involvement and engagement (PPIE) was essential to the design and conduct of ASCEND PLUS.

**Aim:**

To report the process and conduct of PPIE activity in ASCEND PLUS, evaluate effects on trial design, reflect critically on successes and aspects that could have been improved, and identify themes and learning relevant to implementation of PPIE in future trials.

**Methods:**

PPIE activity was coordinated centrally and included six PPIE focus groups and creation of an ASCEND PLUS public advisory group (PAG) during the design phase. Recruitment to these groups was carefully considered to ensure diversity and inclusion, largely consisting of adults living with type 2 diabetes from across the UK. Two members of the PAG also joined the trial Steering Committee. Steering Committee meetings, focus groups, and PAG meetings were conducted online, with two hybrid workshops to discuss PPIE activity and aspects of the trial.

**Results:**

PPIE activity was critical to shaping the design and conduct of ASCEND PLUS. Key examples included supporting choice for participants to either complete the screening/consent process independently online, or during a telephone or video call interview with a research nurse. A concise ‘initial information leaflet’ was developed to be sent with the initial invitations, with the ‘full’ information leaflet sent later to those interested in joining the trial. The PAG reviewed the content and format of participant- and public-facing materials, including written documents, online screening forms, animated videos, and the trial website, to aid clarity and accessibility, and provided input into the choice of instruments to assess quality of life.

**Conclusions:**

PPIE is integral in ASCEND PLUS and will continue throughout the trial. This involvement has been critical to optimising the trial design, successfully obtaining regulatory and ethical approval, and conducting the trial.

**Supplementary Information:**

The online version contains supplementary material available at 10.1186/s13063-024-08393-2.

## Introduction

ASCEND PLUS is an ongoing randomised placebo-controlled trial assessing the effects of oral semaglutide on cardiovascular and other outcomes in people with type 2 diabetes and no history of heart attack or stroke (NCT05441267). ASCEND PLUS will recruit approximately 20,000 participants in the UK. Potential participants are sent an invitation by post and the trial requires no in-person visits. Study medication is mailed directly to participants’ homes. This design represents a shift from the traditional concept of face-to-face interaction between research staff and participants at a clinical site and has become more common in recent years, perhaps accelerated by the COVID-19 pandemic [1]. Decentralised direct-to-participant designs, including that of ASCEND PLUS, offer the possibility to expand participation in clinical trials and increase the generalisability of results [1].

The ASCEND PLUS trial design was developed with extensive patient and public involvement and engagement (PPIE), to ensure that the participant experience is as good as it can be, the safety and wellbeing of the participants is protected, recruitment to the trial is successful, and the engagement and adherence of participants is maintained. ASCEND PLUS commenced recruitment in March 2023, and the estimated primary completion date of the trial is 2028.

PPIE is increasingly recognised as a key element in the development of all research [2], including clinical trial proposals and protocols. PPIE can harness the valuable insights of those living with and affected by a disease or health condition, and ensure that the trial findings are relevant to the needs of patients, and their relatives and carers [3]. “Involvement” can be defined as activities and research carried out “with” or “by” members of the public or patients, rather than “to”, “about”, or “for” them. In this instance, this refers to the active involvement of patients and members of the public in the development of the trial design and the conduct of the trial [4]. In contrast, “engagement” focuses on how the trial findings can be shared with patients and the public in a two-way process that encourages communication and interactions with researchers [4]. Despite the recognition of the importance and potential value of PPIE in clinical trials, implementation remains variable at present with inconsistency between trials [5].

Here, we aim to report the process and details of PPIE activity during the planning and initiation of ASCEND PLUS, evaluate how this helped to shape the final trial design, reflect critically on successes and aspects that could have been improved, and draw out themes and learning relevant to the implementation of PPIE in future trials.

## Methods and results

### Theoretical considerations

The revised Guidance for Reporting the Involvement of Patients and the Public (GRIPP-2) long-form checklist [6] was used to guide the drafting of this report (see Supplementary Table 1).

### Resourcing of PPIE activities

PPIE in ASCEND PLUS was organised by dedicated PPIE officers working within the communications team alongside the core trial team comprised of investigators, trial managers, and administrative staff at the Nuffield Department of Population Health at the University of Oxford (which sponsors the trial). An appropriate level of funding was available in the trial budget for PPIE activity, and all PPIE representatives were able to claim monetary compensation for their time, lived experience, and contribution, in line with guidance from the National Institute for Health and Care Research (NIHR) [7] and accepted best practice. Any out-of-pocket expenses (such as travel) incurred by PPIE representatives were reimbursed in full, and refreshments were provided at in-person meetings.

### Format of PPIE activities

PPIE activity in ASCEND PLUS consisted of several linked components, beginning early in the design phase of the trial and is planned to continue through to trial completion and dissemination of the results.

Firstly, a series of six patient and public focus groups were convened to address specific issues. These focus group meetings largely involved people living with type 2 diabetes and included people from diverse backgrounds from across the UK.

Secondly, a trial-specific Public Advisory Group (PAG) was established. The PAG is responsible for providing feedback, advice, and opinions on many different aspects of ASCEND PLUS over the entire lifecycle of the trial.

Thirdly, in order to ensure patient involvement in the design and conduct of ASCEND PLUS at a strategic level, two members of the PAG who are individuals living with diabetes were also invited to join the Trial Steering Committee.

Steering Committee meetings, focus groups, and PAG meetings were largely conducted online using remote meeting software. Two in-person PPIE workshops were convened in Oxford. This combination of online and in-person meetings has been suggested to be favourable in a previous mixed methods study [8].

### Recruitment and selection of focus groups and the PAG

The recruitment and selection of the focus groups and the PAG was carefully considered to ensure inclusivity and representation, for features including age, sex, and ethnicity. People living with type 2 diabetes were prioritised, given that ASCEND PLUS is a trial in this population.

The six focus groups were organised with support from the Nuffield Department of Population Health’s Public Advisory Group and four external organisations (Table 1). Each focus group was drawn from a specific geographic location (Leicester, Oxford, the north of England [two groups], Wales, and Scotland), to provide coverage of the areas of the UK in which ASCEND PLUS plans to recruit. The focus group based in Leicester was from the Centre for Ethnic Health Research and consisted of individuals of South Asian, Black Caribbean, and Black African ethnicity. The size of, and strategy used to achieve diverse representation within, each group was usually determined by the groups themselves.

**Table 1:** Summary of six focus groups conducted during the development of trial processes

Group (*composition; date*)	Subjects discussed	Feedback (*impact*)
Subset of the Nuffield Department of Population Health departmental Public Advisory Panel, University of Oxford (*10 panel members, some are participants of previous trials; June 2021*)	- Consent model - Recruitment materials - Invitation method (including use of data without consent)	- Proposed a choice of consent methods (self-directed online on own device [with option to speak to a nurse or study doctor] or interview with a research nurse) - Suggested an initial information leaflet with the invitation and further information sent after (*implemented*) - Support for use of health data in this way but with a focus on transparency
Research for the Future^a^: Group 1 (*8 people living with diabetes from the north of England; July 2021*)	- Consent model - Run-in treatment - Recruitment materials - Invitation method (including use of data without consent)	- Support for a choice of consent methods - Suggested an animation or film to make the study easy to understand (*implemented*) - Agreement with the colour coding of run-in treatment bottles. Suggested to add text- e.g. “take this first” (*partially implemented – unable to add to label, added to treatment information leaflet)* - Supported an initial leaflet first and then the full participant information leaflet (PIL) - Support for use of health data for invitation. Recommended adding detail on approvals required (*implemented*)
Research for the Future^a^: Group 2 (*11 people with diabetes from the North of England; July 2021*)	- Consent model - Run-in treatment - Recruitment materials - Invitation method (including use of data without consent)	- Support for a choice of consent methods - Needs to be clear in the PIL that everyone takes the active drug during the run-in (*implemented*) - Supported an initial leaflet first and then the full PIL - Rationale for including people whose blood sugar levels are already well controlled needs to be clear (*implemented*) - No concerns about invitation method (but limited time to discuss this point)
Diabetes Research Group, Swansea (*5 people with diabetes from Wales; Sept 2021*)	- Consent model - Invitation method (including use of data without consent)	- Support for a choice of consent methods - Concern about how a letter from the University of Oxford might be received (*discussion initiated with Digital Health and Care Wales to investigate possibility of letters sent from within the NHS)*
Diabetes Scotland (*7 people with diabetes from Scotland; Sept 2021*)	- Consent model - Invitation method (including use of data without consent)	- Support for choice of consent method. The option of speaking to the research team needs to be clearly available throughout (*implemented*) - Support for use of data for invitation but suggestion that including someone more local on the invitation letter may be better received (*under consideration)*
Centre for Ethnic Health Research, University of Leicester (*6 people with diabetes from Asian or Black backgrounds; October 2021*)	- Recruitment materials - Consent model	- Involve local groups/trusted community leaders at recruitment stage—could be to raise awareness rather than actually recruit to the trial (*under consideration)* - Support for choice of consent methods for English speakers but concern about how well non-English speakers might understand translated online content, so recommended nurse interviews with translation for these individuals. Additional risk from inaccuracies in translation. Suggested telephone or video call for non-English speakers (*implemented*)

^a^Research for the Future is a NIHR CRN (National Institute for Health and Care Research Clinical Research Network) initiative to help recruit people to take part in health and care research using a ‘consent for approach’ model

Members of the PAG were invited from an existing departmental public advisory panel and the focus groups described above. The PAG was chosen to comprise a diverse group of patients and the public.

### Involvement of PPIE panels and the PPIE advisory group in the research proposal

During the design phase of ASCEND PLUS, the six online PPIE focus groups (described above) were convened to address specific issues. Given the remote design of the trial with no in-person visits, the main topics discussed were the consent model and the recruitment/invitation methods. There was also discussion about other aspects of the trial design, including the active run-in, in which all participants receive the active drug prior to randomisation. These concepts were serially developed across the six focus groups, which took place between June and September 2021, with revisions made to the study design in response to the feedback received prior to the application for ethical approval.

Two people living with diabetes were next invited to join the Steering Committee. These individuals attended the first Steering Committee meeting in June 2021 and will continue to attend Steering Committee meetings until the completion of the trial. These patient and public contributors are members of the Steering Committee, contribute to discussions at meetings, and can vote on any decisions made by the Committee. They are also the joint senior authors of this publication (SD and JR).

The trial PAG was then assembled, including SD and JR amongst the members. The PAG contributed in detail to the design and review of all patient-facing study material, the online forms and videos used for the trial, and the trial website. This activity was organised through emails and online group meetings, as well as one face-to-face workshop in Oxford. The PAG will continue to contribute during the remainder of the trial, for example by reviewing planned patient newsletters and advising on local activities to aid recruitment. The PAG will also input on the interpretation of the trial results in due course, and specifically on their presentation and dissemination to patients and the public.

### Impact of PPIE on ASCEND PLUS design and conduct

The six PPIE focus groups were critical to shaping the design and conduct of ASCEND PLUS. Full details of the composition and date of each of the six focus groups, the subjects discussed, and the feedback and impact are summarised in Table 1.

Initially, it had been planned to invite all participants to complete self-directed online screening and consent, with an option of a telephone or video call if needed. A clear theme that emerged in the focus groups was support for choice in how participants interact with the trial: i.e. either online completion of study assessments on their own device (with the option to speak to a research nurse or study doctor at any time) or completion of study assessments during interviews with a research nurse. Therefore, recording of informed consent also needed to include both an online consent option (that can be completed by a participant independently) and the option to give consent during a telephone or video call with a research nurse. It was also felt to be important that participants can switch between these two methods of participation at any stage if they wish to. The exception to this concept was for non-English speakers, in whom a telephone or video call with a research nurse (aided by a translator) was recommended to ensure adequate understanding. In light of this feedback from the focus groups, the trial procedures were modified. The updated trial design now asks potential participants to indicate on the initial reply form which method (self-directed online versus telephone/video call with a research nurse) they prefer. Options have also been added to allow participants to change their trial interaction method during the course of the trial.

Another key impact on design and conduct of the trial resulted from feedback that the patient information leaflet was very long, due to the need to contain multiple items deemed mandatory by regulatory bodies. The focus group supported provision of an abbreviated “initial information leaflet” (rather than the “full” participant information leaflet) with the invitation letter, with the “full” patient information leaflet [9] subsequently supplied to those individuals who had declared interest in participating after reviewing the abbreviated leaflet.

The PPIE focus groups supported the proposed invitation method for ASCEND PLUS. In brief, this is conducted with the support of the NHS DigiTrials recruitment support service who undertake a search of electronic medical records to identify individuals who are potentially eligible (without individual patient consent at this stage). The name, address, and postcode of these individuals are then passed securely to a mailing house (who also handle patient letters for the NHS) who then send out study invitation letters. The details of potential participants are not disclosed to the ASCEND PLUS study team unless and until the participant returns the reply form, which includes the participant’s name and the details they add to it (such as telephone number, or email address). The reply form also contains a unique identifier which the ASCEND PLUS team send to NHS DigiTrials to obtain the participant’s name, address, sex, date of birth, NHS number, and GP surgery details from NHS records. The positive feedback from the PPI focus groups regarding the use of healthcare data in this way was cited in the application for regulatory approval. This recruitment method was supported by the Health Research Authority (HRA), who also followed advice from the Confidentiality Advisory Group (an independent body which provides expert advice on the use of confidential patient information). A separate data protection leaflet which is supplied to prospective participants covers all aspects of how data about ASCEND PLUS participants is processed [10].

**Fig. 1:**
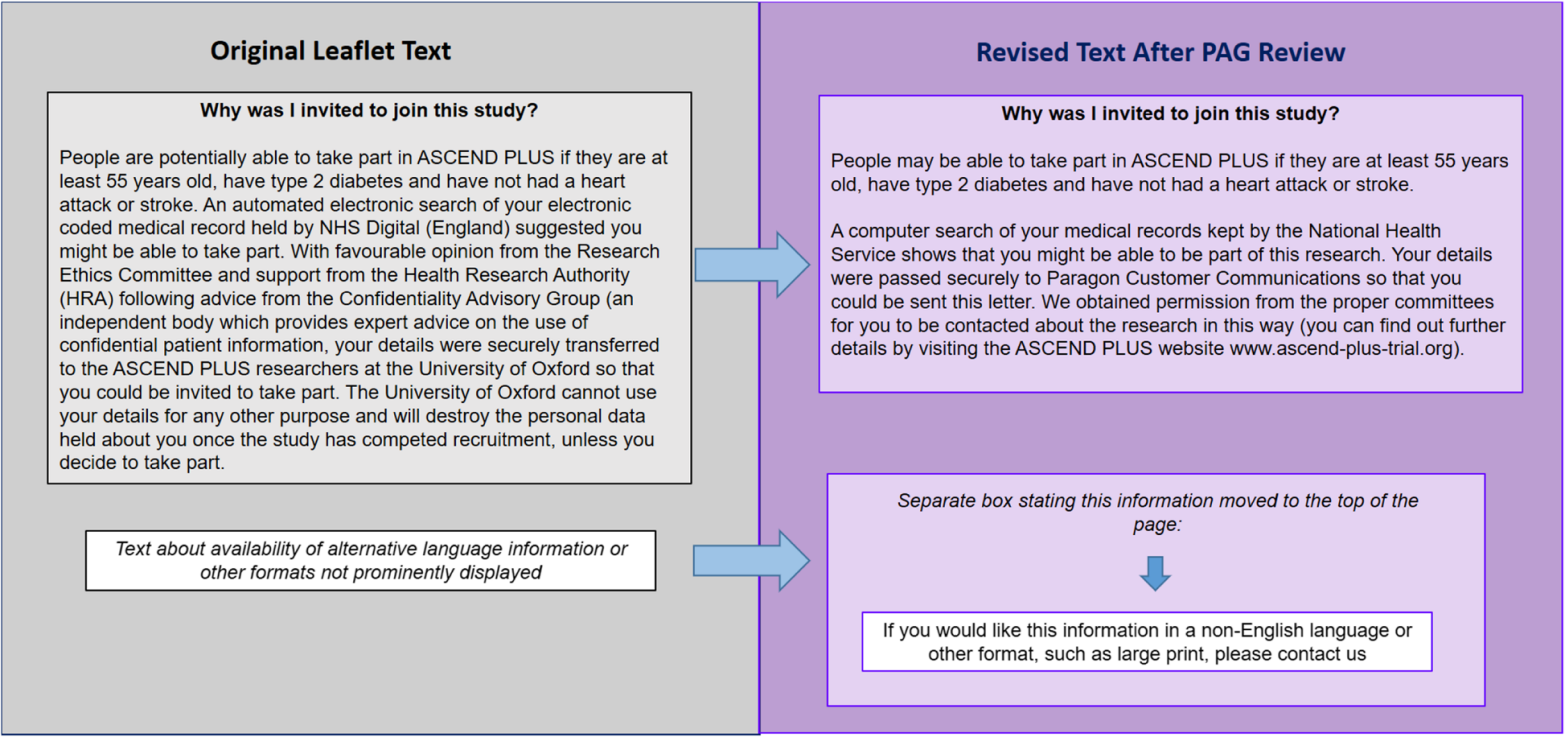
Examples of specific changes made to the text of the ASCEND PLUS participant information leaflet after PPIE input

The ASCEND PLUS PAG also undertook a detailed review of the three leaflets discussed above (initial information leaflet, full participant information leaflet, data protection leaflet), the trial invitation letter, and the study treatment information leaflets (one of which is included with each pack of study treatment mailed to a participant). Recommended text and content changes were made accordingly, ensuring that the text of each document remained consistent with trial processes. This extensive PPIE review and consultation process has resulted in documents which are easier to understand and more inclusive. This also included feedback about accommodating people with visual impairments. Examples of specific changes made to the text of study documents are shown in Fig. 1.

The PAG were then involved in co-developing an animated video to support the self-directed online consent process. The PAG initially contributed to the development of the script and then provided feedback on the images used in the storyboard, with many of the specific points raised implemented in the final version. For example, the images of potential participants in the video were updated to ensure greater diversity, and a border line was drawn on a map of the UK to highlight the geographical areas in which ASCEND PLUS plans to recruit.

The PAG was instrumental in the selection of quality of life questionnaires included in ASCEND PLUS. Whilst inclusion of the EQ5D questionnaire is commonplace due to its importance to the National Institute for Health and Care Excellence (NICE), several options existed for an additional questionnaire to capture diabetes-specific quality of life. The Problem Areas in Diabetes (PAID), Patient Health Questionnaire 9 (PHQ-9), Diabetes-Dependent Quality of Life questionnaire (ADDQoL), and the Diabetes Treatment Satisfaction Questionnaire (DTSQ) were all considered. PAG members ranked the questionnaires separately on whether they thought they collected a meaningful and relevant assessment for people with diabetes, and whether participants would be willing to complete them. The PAG members were also separately asked to consider the feasibility of participants completing the 36-Item Short Form Survey questionnaire (SF-36) compared to the 12-item version (SF-12). Following detailed feedback from the PAG, the SF-12 and the PAID questionnaire were included in the final ASCEND PLUS protocol.

The PAG also reviewed the text and format of the questions included in the draft screening form (to be completed either by participants on their own devices or by research nurses in conversation with participants) and provided detailed feedback. A number of changes were implemented based on this, including changes to the order in which questions are asked and revisions to the working of particular questions to make them easier to understand.

**Fig. 2:**
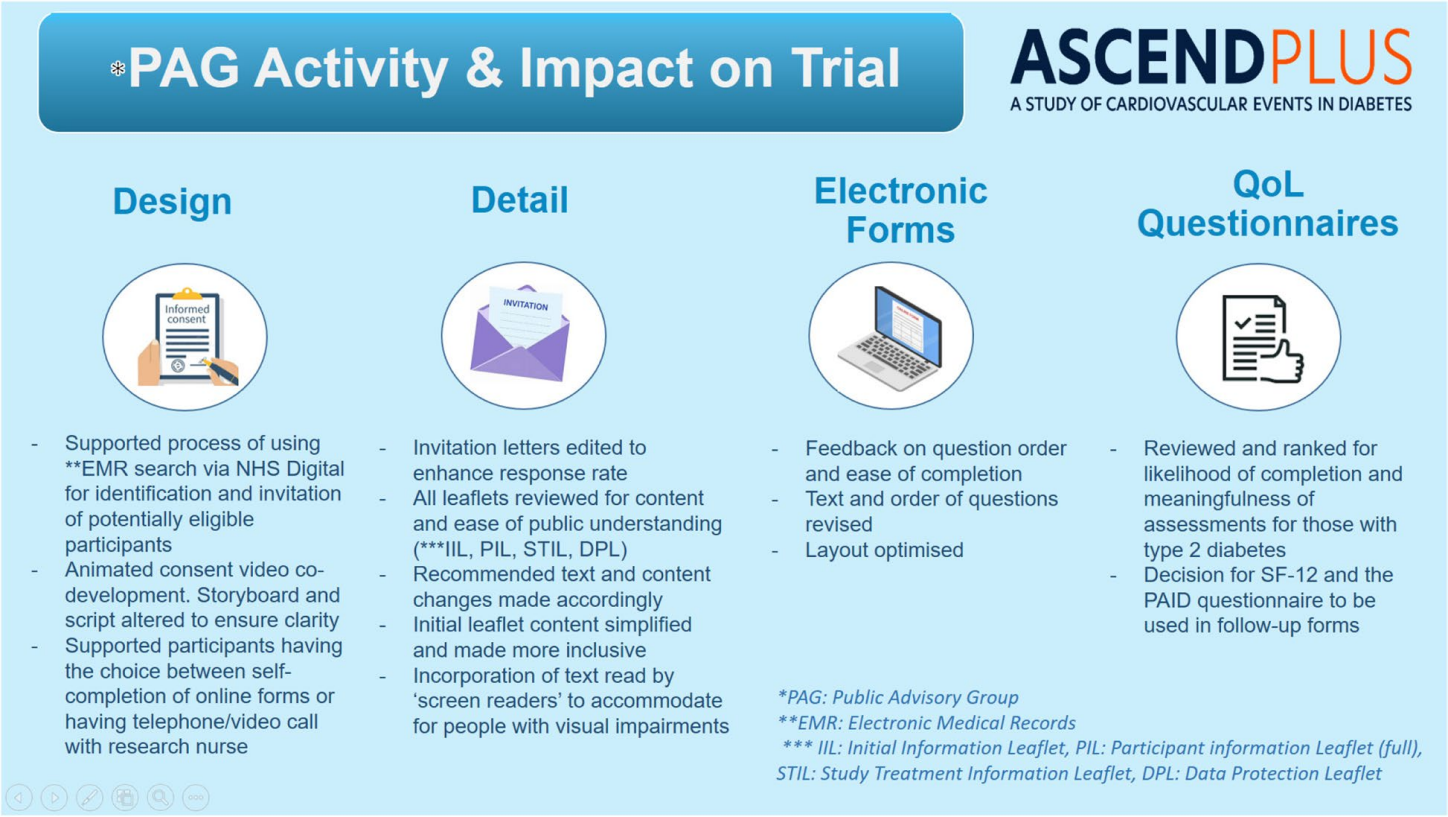
Summary of Public Advisory Group activity and impact in ASCEND PLUS

A summary of PPIE in ASCEND PLUS is included in a dedicated page on the trial website, which also includes a video of two public contributors discussing their experience [11]. This activity, and the impact that it has had on the final design of ASCEND PLUS, is also summarised in Fig. 2.

### Impact of PPIE on individuals involved, and wider impact

The impact of PPIE in ASCEND PLUS on the individuals involved and the wider impact was considered in detail at a workshop convened on 26 November 2022, which included ten members of the PAG as well as investigators, trial managers, research fellows, and PPIE officers from the Nuffield Department of Population Health.

The context and process of PPIE in ASCEND PLUS were considered in some detail. Themes that emerged in this discussion included the fact that PAG members reported an overall highly positive experience. They commented that on-boarding for new members worked well and that the process had been well organised, with all members having a clear idea of upcoming tasks with regular updates from the study team. Having a single point of contact (the PPIE team at the Nuffield Department of Population Health) to coordinate the PPIE for the study was felt to be a major advantage. The logistical aspects of PAG group meetings were discussed and the format of online meetings scheduled in the evenings or at weekends was felt to be beneficial in avoiding travel time and allowing individuals the flexibility of contributing from their own home. Many meetings took place during periods of COVID-19 lockdowns, and being able to hold online meetings enabled these to go ahead and brought people together at a time of isolation. The benefits of in-person events (such as the PPIE workshop) were also discussed, and it was felt that some aspects, such as the ability to arrive early for social discussion and remain behind after the main meeting to ask questions and have private conversations, could also be implemented using the existing features offered by major online meeting platforms.

The inclusive nature of the PPIE process in ASCEND PLUS was praised. Specifically, discussion focused on the decision to actively encourage the involvement of contributors without previous PPIE experience, as well as the expectation in PAG meetings that everyone is listened to equally and that there are “no silly questions”. Members of the PAG also reflected that external feedback on ASCEND PLUS documents (created with their input) has been very positive. For example, the Departmental Information Governance lead commented that the ASCEND PLUS data information leaflet was the best such example they had seen in their experience of advising on multiple trials over a number of years. Similarly, the process of conducting PPIE in ASCEND PLUS has been used as an exemplar in the MSc in Clinical Trials postgraduate course that is run by the Nuffield Department of Population Health at the University of Oxford for students from across the world.

Some areas that could have been improved were also identified. Occasionally, too much information could be presented in PAG meetings, and key questions cropping up towards the end of a meeting might have meant that they received less attention than they should have. It was also highlighted that technology can be a barrier for some people, particularly those lacking the digital skills or hardware to be able to participate in online meetings. For example, printed materials may need to be offered as not all individuals will have access to a printer. In a few cases, deadlines for responding to tasks were shorter than ideal, and it was recommended that circulation of slides and materials should be undertaken well in advance of a meeting to allow members enough time to consider them carefully. Finally, it was suggested that it would have been helpful to have a “global overview” of the planned PAG activities so that members had an idea of what had been completed already and what would be coming up next.

In terms of the effect on themselves as individuals, PAG members reported that they had found participation in PPIE activities for ASCEND PLUS highly enjoyable and reported that there was more “behind-the-scenes” activity than they had initially expected. There was consensus that it was highly rewarding being part of helping to create a study that may have a huge impact on people’s lives, and in ensuring that the study is accessible to people from all walks of life including groups who are traditionally under-represented in research. From a personal perspective, some members reported that they had found participation intellectually stimulating and that it helped them to keep up to date with diabetes research, and be more confident when talking about research in general.

Finally, it is recognised that this manuscript only presents qualitative reflections on PPIE in ASCEND PLUS. ASCEND PLUS is still early in recruitment at the present time, and presentation of quantitative data on recruitment and retention would not be particularly meaningful in the absence of a relevant control. Of note, a sub-study is planned to specifically evaluate consent in ASCEND PLUS, given the decentralised trial design.

## Discussion

### Study design of ASCEND PLUS and the relevance to the PPIE strategy

At the outset of ASCEND PLUS, a number of challenges and opportunities were identified that would be critical to the success of the trial, including:

1. Gaining approval from the relevant bodies for an innovative, streamlined trial design that has no in-person visits and requires a non-traditional participant consent process.
2. Recruitment of a large number of people (20,000), aged 55 and over, living with type 2 diabetes from across the UK, who have not yet experienced a heart attack or stroke. In addition, ethnic diversity among trial participants is highly desirable, to ensure a trial population broadly representative of that of the wider UK.
3. Implementation of a trial where all interactions with participants would be conducted directly using innovative patient-centred web-based technology, supplemented by telephone, video-call contact and mailed letters.
4. A decentralised enrolment and consent process that is sufficiently flexible and adaptable to suit all participants, irrespective of preference for self-directed online interaction versus a telephone/video call with a research nurse.
5. A lengthy participation timescale of 5 years.

The role of PPIE was particularly critical in ASCEND PLUS, helping to optimise the trial design in the context of each of these points. As discussed in the above sections, various aspects of the trial design were altered in line with the public contributors’ feedback, sometimes in quite a major way such as the decision to allow choice in the method of interaction with the study.

### Contextual and process factors influencing PPIE in ASCEND PLUS

PPIE in ASCEND PLUS has included several distinct phases, including six focus groups, the construction of a trial-specific PAG, and inclusion of two members of the PAG on the trial Steering Committee.

PPIE activity in ASCEND PLUS has been greatly enhanced by the recruitment of enthusiastic and dedicated members to the focus groups and PAG, coordination and organisation by experienced and professional PPIE officers, the willingness and desire of the trial investigators to modify the trial design in response to PPIE feedback, and adequate resourcing for PPIE activity in the trial budget. The use of digital technology and online meetings aided the efficiency and inclusivity of the process.

Some areas of difficulty were identified. The tight trial timeline meant that occasionally PAG members were under pressure to meet challenging deadlines for review of various materials, and some online sessions perhaps contained too much information. Adequate resourcing of PPIE activity is key to spreading the load on each individual member.

### Influence of PPIE on the final ASCEND PLUS study design

PPIE greatly enhanced the final ASCEND PLUS study design. The changes made in response to the PPIE scoping exercises made the trial more inclusive, most notably in influencing the decision to give all participants a free choice in the method by which they interact with the study. The PPIE activity also heavily influenced almost all of the written and online material for the trial, making this more accessible and understandable, and also available in different formats and to those with visual impairment. Specifically, the experience afforded by individuals living with diabetes was highly valuable when considering the nature of the trial and the target population. The impact of PPIE activity on the study was evaluated by direct comparison of the final trial design to the initial proposals (with some examples included here), and by qualitative discussion with relevant stakeholders in a dedicated workshop convened for this purpose.

### Learnings and recommendations for future large-scale clinical trials

**Fig. 3:**
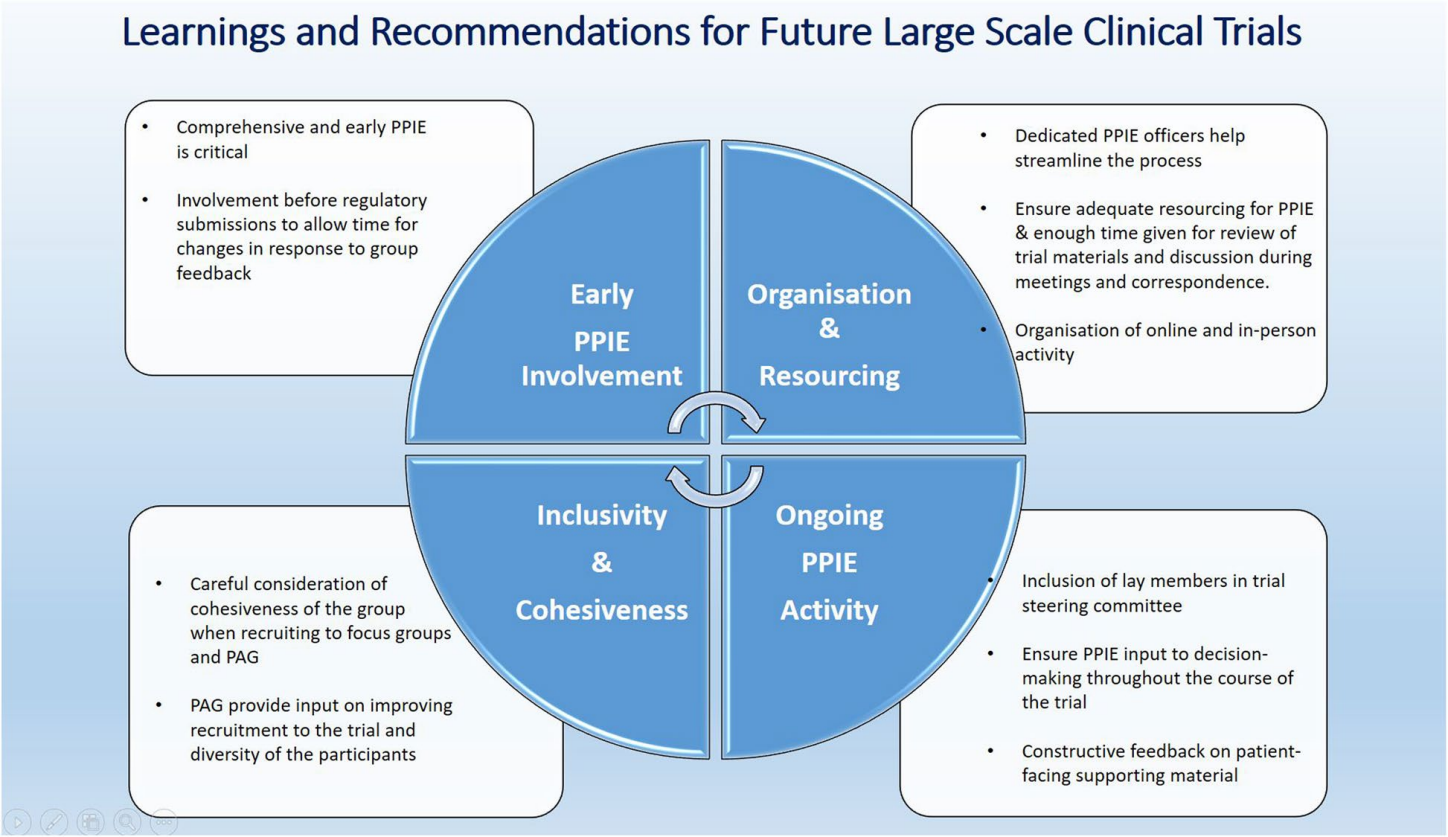
Learnings and recommendations for PPIE in future large-scale clinical trials

Comprehensive and early PPIE is critical to gain input on all aspects of the proposed trial design and to optimise the relevance and acceptability to people living with diabetes. This involvement should start well before regulatory submissions, in order to allow time for changes to be made in response to PPIE group feedback. Involvement of dedicated and professional PPIE officers should be strongly considered to streamline the process, and adequate resourcing of PPIE activity is essential. Careful consideration should be given to how recruitment to focus groups and advisory groups is undertaken, making sure that assembled panels are inclusive and representative, and are able to work in a cohesive group and provide constructive comments and feedback in a timely manner. Feedback on participant- and public-facing supporting material such as information leaflets, animations, and the trial website helps to make these accessible and should improve recruitment and adherence, as well as the experience of recruited individuals. Inclusion of public contributors on the trial Steering Committee is important to ensure PPIE input to decision-making during the course of the trial. A summary of these recommendations is outlined in Fig. 3. Finally, ASCEND PLUS is a UK-based trial, and there may be limited applicability to different healthcare systems and cultural contexts, or in resource-limited settings.

## Conclusions

ASCEND PLUS is a large-scale trial with an innovative, streamlined design with a non-traditional participant consent process and no in-person study visits. Extensive PPIE has proven integral to the design and initiation of ASCEND PLUS and will continue throughout the trial. This involvement has been critical to optimising the trial design, successfully obtaining ethical and regulatory approvals, and conducting the trial.

### Supplementary Information

Supplementary Material 1. Table 1: GRIPP-2 checklist.

## Data Availability

Not applicable.
